# The COVID-19 Impact and Culture Nexus in Japan: Insights for the Global Community

**DOI:** 10.3389/fpubh.2022.879653

**Published:** 2022-05-09

**Authors:** Cornelius B. Pratt, Ronald Lee Carr

**Affiliations:** ^1^Communication Psychology and Application Research Center, Northwest University of Politics and Law, Xi'an, China; ^2^Lew Klein College of Media and Communication, Temple University, Philadelphia, PA, United States; ^3^Department of Communication Studies, Temple University Japan, Tokyo, Japan

**Keywords:** COVID-19, Japan, sociocultural factors, protesters, endemicity

## Introduction

This opinion column identifies and reflects on the defining moments in Japan's sprint toward acquiring herd immunity by the end of 2021. As of this writing, COVID-19 conversations across the globe are becoming less and less concerning, as wealthy nations ramp up testing and triple-vaccinate their citizens, as COVID-19-related infection and hospitalization rates fall significantly, or, as is the case of the less developed countries of the Global South, concerted efforts are being made to have a sizable number of residents tested for COVID-19 and inoculated with their first shot. In developed economies, the pendulum of healthcare uneasiness is pivoting toward a simple issue—endemicity—as governments ease or phase out COVID-19 restrictions, as the disease is increasingly being viewed through the lens of, say, the seasonal flu, and as agitation against COVID-19 mandates leads to protests in countries such as the Netherlands, France and Canada. The world will get by, the argument goes, by coexisting with COVID-19 as it does with a host of other diseases, including the seasonal influenza, dengue, and malaria. But applying such a view now to the pandemic may be inadvisable, as Tedros Adhanom Ghebreyesus, director general of the World Health Organization, asserts:

“*We're concerned that a narrative has taken hold in some countries that because of vaccines, and because of Omicron's high transmissibility and lower severity, preventing transmission is no longer possible and no longer necessary. Nothing could be further from the truth. More transmission means more disease* (1).”

But the world need not have been so ravaged by the pandemic only if it had looked around to remind itself about commonsensical steps that could have been considered in response.

## Reflections on Defining Outcomes

In this opinion, we reflect on some of the defining actions that steered the island nation toward managing effectively its own response to COVID-19. To do so, we consider insights into tactical and sociocultural perspectives on that question. We consider factors that can serve as an eye-opener to the international community in its battle against a global scourge. But, first, a backdrop on some of the key issues that have bedeviled the public-health community.

As of March 31, 2022, worldwide, there were more than 485 million cases of COVID-19 and more than 6.14 million deaths from it, making it a major global health issue. In Japan, the effects of COVID-19 are not limited to clinical settings; the disease has had clear implications for the country's political outcomes. Yoshihide Suga, Japan's former prime minister, left office in just 1 year, following criticisms leveled at his government for its initially slow rollout of COVID-19 vaccines, the resulting spikes in COVID-19 infections, and the fallout from his government's refusal to approve compensation for economic injury suffered by furloughed workers because of a slump in the business sector. In national elections held early in October 2021, his successor, Fumio Kishida, of the same Liberal Democratic Party, won a clear majority in Japan's lower, more powerful parliamentary chamber, signaling a refreshing resolve by Japan to reduce significantly chances for any uptick in the spread of the coronavirus, thus placing the country firmly on a trajectory toward achieving herd immunity.

Japan was mired in disputes over hosting the summer 2020 Olympic Games. The cost of hosting the rescheduled games, estimated at nearly $14 billion, loomed large on the minds of both the Japanese public, who were excluded from all spectator stands at the games, and of the organizers. About 1 week to kickoff, 78% of respondents in Japan thought COVID-19 concerns justified postponing the games at the very least (2), even as additional questions were raised on what the new COVID-19 precautions, the 1-year postponement of the games, and the atmospheric-ocean dynamics in Tokyo in summer 2021 will “*mean for preparedness efforts for the athletes, coaches, clinicians, and volunteers* (3).” Months before the start of the games, Japan had such low caseloads of COVID-19 infections that some European countries looked languid in their strategic responses to the virus.

As of this writing, with more than 75% of its population having received the second dose of COVID-19 vaccine, and with more than 33% having received a booster shot, the 45,112 average daily new infection cases are still among the lowest among industrialized nations, justifying concluding that Japan now has herd immunity, which occurs when a large enough segment of a population becomes immune to a disease, making its spread less likely.

COVID-19 deaths per capita, by country, show Japan has the lowest rate among industrialized countries, except New Zealand, whose modest casualty statistics–28 deaths and 5.69 deaths per million inhabitants (2, 4)—offer the global community some reassurance of the possibility of nixing a vexing health issue. As of February 2022, Japan had more than 3.3 million confirmed cases, 19,341 deaths, 144.5 deaths per million inhabitants (or 14.47 per 100,000). Statistics from the Ministry of Health, Labor and Welfare show that while Japan's number of newly confirmed daily cases has risen to an average of 89.489 in February 2022, the number of severe cases has dropped by half compared to the number in September 2021 (5). Another reassuring trend is in the comparison of the age of positive cases with that of severe cases and morbidity: As of February 2022, the highest age range for new COVID-19 cases for males and females was 20–29 years, yet, the highest number of severe cases and mortality was among males older than 70 years. Among Japanese children, the trends have also been encouraging: During the Delta wave (August-September 2021), the number of children (1.4% of COVID-19 admissions to intensive care units) treated for COVID-19 infections was 14 times higher than was the case before the Delta surge between October 2020 and May 2021 (6). During periods before and after the Delta wave, there were no reported deaths among infected children.

In comparison, the United States has much higher rates of severe cases and mortality among children. During 2 weeks, January 27, 2022, through February 10, 2022, there was an 8.2% increase in the cumulative number of child COVID-19 cases since the beginning of the pandemic, and that in states reporting, between 0.00% and 0.01% of all child COVID-19 cases resulted in death (7). Comparatively, Japan has actively managed the impact of the coronavirus on children and young adults.

Early in the pandemic, Japan used a cluster-focused approach based on its understanding of the transmission of the virus. This approach revealed “*that in most cases a majority of patients did not infect others, but that a limited number of cases caused more than five secondary cases, forming clusters* (8).” The government used those clusters to learn more about environmental risk factors and behaviors, to track down unidentified cases, to issue public warnings about transmissions, and to justify publicly its campaign slogan against gatherings—that is, urging the public to eschew “closed, crowded spaces with close-contact (the three Cs).”

That strategy to mitigate viral transmission in the population seemed effective. However, a concerning reality of COVID-19 has been the fragility of public-health systems, particularly those in developed economies, even as their resilience has never been in doubt. At the peak of the incidence of COVID-19 in Japan, for instance, it lagged most other developed nations in terms of the percentage of its citizens who had received at least one shot of any vaccine against the virus. Underpinning that vaccination drive is the uneven global access to vaccines, a situation the World Health Organization described in September 2021 as unacceptable.

## Five Contributory Factors

In this column, we reflect on five factors that have contributed to pivoting the country on a trajectory toward accomplishing the much-desired herd immunity to the disease—indicating a bold attempt by Japan to nudge itself back on track, following a few missteps, toward acquiring herd immunity.

One of the challenges of global efforts toward reaching herd immunity in the battle against COVID-19 is encouraging—if not persuading—the public to take a significant, yet a simple, step: get inoculated. Reflecting on the role of five factors in Japanese society can help public-health experts demystify the undercurrents of Japan's successful race to herd immunity. The first of such forces is the government's clear, consistent, forthright public announcements on an antidote to the health crisis. (To ensure consistency in COVID-19 messaging, only the national government, through the Ministry of Health, Labor and Welfare, makes countrywide announcements on the virus). For example, on June 8, 2021, Taro Kono, Japan's minister charged with managing COVID-19, delivered to the nation a 2 min, 21 S speech in which he said: “*While side reactions, including localized pain and fever do occur, the government recommends that people get vaccinated because the benefits of having the vaccine outweigh the drawbacks of side effects* (9).”

Other national-government talking points, crafted to “provide the information clearly and precisely,” use consistently the same two-word grammatical subject italicized here:

- *COVID-19 vaccines* are effective in reducing your risk of developing symptoms.
- *COVID-19 vaccines* will benefit you, as they are essential to reducing the burdens on medical institutions.
- *COVID-19 vaccines* will be available free of charge.
- *The Government* recommends that people get vaccinated because the benefits of vaccination are greater than the risk of side effects. (Minister Kono also used this talking point in his national broadcast on June 8, 2021.)
- *The Government* will continually confirm the safety of the vaccines and provide safety-related information.
- *The Government* is working in an all-out manner to expedite vaccinations.

In the United States, however, there is some inconsistency in COVID-19-related messages disseminated by the Centers for Disease Control and Prevention (CDC) (10). o much public concern had been expressed on the abstruseness of the U.S. federal government's COVID-related announcements that Ned Lamont, governor of Connecticut, said on national television on November 18, 2021: The “*CDC speaks Latin. I can't figure out who is eligible [for a booster shot], who's not eligible* (11).” As Simona Georgescu, a crisis communication expert, said, “… *the White House and CDC could have avoided what is now widely considered mixed messages and constantly changing recommendations that impacted public trust and potentially damaged public adherence to new guidelines and recommendations* (12).”

The second is the absence of media reportage of divisive misinformation and antithetical actions from publicly avowed anti-vaccine protesters. That absence at the media, audience and agitator levels may be a response to the absence of ukases and mandates—for example, on lockdowns and on vaccine inoculation—that have plagued several nations in both the Global North and the Global South. Moreover, it is plausible to conclude that such absence presents optics of cohesive, non-distractive messages about the efficacy of COVID-19 vaccines and why the public can benefit immensely from them.

The third reflects the enormous influence of Japanese organizational management patterns on its ability to sprint toward acquiring herd immunity. Japan's management style contributes to its organizational success. Open, primarily face-to-face communication minimizes communication barriers whereas interdepartmental cooperation and dependency guide action toward accomplishing goals, paving the way for operational effectiveness (13). As Fukuhara notes, the “Japanese people have a high group cohesiveness and tacit consensus with one another” (14), a norm that offers employees a sense of belonging to an organization; of superiors trusting and empowering employees to make decisions; and of management, including that in public administration, encouraging employees to act flexibly.

Thus, implementing a strategic plan can draw upon the resources ensconced in employee innovation. Regarding COVID-19 management tactics, on the one hand, the national government canceled public events and issued several states of emergency, but it did not have any strict lockdowns. On the other, its initial laissez-faire viral-control measures on domestic variants fueled local transmission and its “*reckless relaxation of the border… not only allowed powerful variants from abroad to enter the country but also led to the transfer of variants* (15).”

The fourth, which resonates with the preceding factor, is a combination of social harmony, cooperation and conformity, which, in Japan, is an important cultural virtue that may explain the country's overwhelming public support for, and compliance with, its COVID-19 vaccination program. Citizens are willing to sacrifice self-autonomy for the national good.

Even in countries with a widespread availability of vaccines, there has been a massive resistance to getting the jab. Japan is an exception, but not because of its collectivistic attribute. It must be noted here that, on the one hand, the common view that Japan is a collectivistic society whereas the United States is individualistic has not been empirically substantiated (16). On the other, consistent with modernization theory—which posits the rise of individualism in both Japan and the United States because of economic growth and modernization—collectivism, which subordinates individual goals to family, tribal, organizational or national goals, persists in highly developed East Asian societies such as modern Japan, posing a challenge to modernization theory (17). In response to the inconclusiveness of research findings on that sociocultural binary, Vignoles argues that, because that widely accepted binary culture seems oversimplified and is of limited value, emphasis should be placed on, say, defining concepts more precisely and expanding investigations of cultural identities and stereotypes (18).

The fifth is the public demonstration of how the national interest takes precedence over individual or tribal interest. As a matter of habit, the Japanese wear face masks during flu season to reduce their chances of infecting others rather than becoming infected. This act of preventing infections to others exemplifies the selfless behaviors of the Japanese, contributing to the low number of COVID-19 cases and related fatalities. Japan's approach to catastrophes such as the March 11, 2011, triple-disaster reflected a cultural policy of a cohesive, united front among the Diet, municipalities, and ward offices across Tokyo and northern Japan. Shortly after March 11, the Tokyo Metropolitan Government imposed conservation limits for energy use throughout Tokyo. As a result, ward offices across Tokyo closed their pools, limited the routine use of electricity, and shut down elevators and escalators across the city. Not only did Tokyo wards meet the limits, they also exceeded them.

At bottom, all five factors reflect, to a significant degree, the Japanese culture of monochromatism in compliance with institutional mandates, and that of societal uneasiness with the public behaviors of non-Japanese nationals.

## Conclusion

The summer 2020 Olympic Games tested Japan's resolve in ensuring the safety of athletes, of visitors, of residents. That was accomplished. Extraordinary measures—for example, excluding most spectators from events and constantly monitoring athletes' health—kept the citywide COVID-19 infection rate low. For residents, however, the coalescence of the strategic and the sociocultural explains Japan's effective management of the impact of COVID-19, even as its missteps served as a road map for a better control of the pandemic within its borders.

## Author Contributions

Both authors listed have made a substantial, direct, and intellectual contribution to the work and approved it for publication.

## Funding

This study was funded by the Research Division of Temple University Japan.

## Conflict of Interest

The authors declare that the research was conducted in the absence of any commercial or financial relationships that could be construed as a potential conflict of interest.

## Publisher's Note

All claims expressed in this article are solely those of the authors and do not necessarily represent those of their affiliated organizations, or those of the publisher, the editors and the reviewers. Any product that may be evaluated in this article, or claim that may be made by its manufacturer, is not guaranteed or endorsed by the publisher.
